# Development and Characterization of a Hydrogel Containing Curcumin-Loaded Nanoemulsion for Enhanced In Vitro Antibacteria and In Vivo Wound Healing

**DOI:** 10.3390/molecules28176433

**Published:** 2023-09-04

**Authors:** Thi Thanh Ngoc Le, Thi Kieu Nhi Nguyen, Van Minh Nguyen, Thi Cam Minh Dao, Hoai Bao Chau Nguyen, Cong Thuan Dang, Thi Bao Chi Le, Thi Khanh Linh Nguyen, Phuong Thao Tien Nguyen, Le Hoang Nam Dang, Van Minh Doan, Hoang Nhan Ho

**Affiliations:** 1 Faculty of Pharmacy, University of Medicine and Pharmacy, Hue University, 6 Ngo Quyen, Hue 530000, Thua Thien Hue, Vietnam; lttngoc@hueuni.edu.vn (T.T.N.L.); ntknhi@huemed-univ.edu.vn (T.K.N.N.); dtcminh@huemed-univ.edu.vn (T.C.M.D.); nhbchau@huemed-univ.edu.vn (H.B.C.N.); 2 Faculty of Odonto-Stomatology, University of Medicine and Pharmacy, Hue University, 6 Ngo Quyen, Hue 530000, Thua Thien Hue, Vietnam; nvminh.rhm@huemed-univ.edu.vn; 3 Department of Histology, Embryology, Pathology, and Forensic, University of Medicine and Pharmacy, Hue University, 6 Ngo Quyen, Hue 530000, Thua Thien Hue, Vietnam; dcthuan@huemed-univ.edu.vn (C.T.D.); npttien@huemed-univ.edu.vn (P.T.T.N.); 4 Department of Microbiology, University of Medicine and Pharmacy, Hue University, 6 Ngo Quyen, Hue 530000, Thua Thien Hue, Vietnam; ltbchi@huemed-univ.edu.vn (T.B.C.L.); ntklinh@huemed-univ.edu.vn (T.K.L.N.); 5 Department of Anatomy and Surgical Training, University of Medicine and Pharmacy, Hue University, 6 Ngo Quyen, Hue 530000, Thua Thien Hue, Vietnam; dlhnam@huemed-univ.edu.vn; 6 Faculty of Traditional Medicine, University of Medicine and Pharmacy, Hue University, 6 Ngo Quyen, Hue 530000, Thua Thien Hue, Vietnam; dvminh@huemed-univ.edu.vn

**Keywords:** curcumin, nanoemulsion, optimization, wound healing, gel

## Abstract

Curcumin (CUR) is a natural compound extracted from turmeric (*Curcuma longa* L.) used to cure acne, wound healing, etc. Its disadvantages, such as poor solubility and permeability, limit its efficacy. Nanoemulsion (NE)-based drug delivery systems have gained popularity due to their advantages. This study aimed to optimize a CUR-NE-based gel and evaluate its physicochemical and biological properties. A NE was prepared using the catastrophic phase inversion method and optimized using the Design Expert 12.0 software. The CUR-NE gel was characterized in terms of visual appearance, pH, drug release, antibacterial and wound healing effects. The optimal formulation contained CUR, Capryol 90 (oil), Labrasol:Cremophor RH40 (1:1) (surfactants), propylene glycol (co-surfactant), and water. The NE had a droplet size of 22.87 nm and a polydispersity index of 0.348. The obtained CUR-NE gel had a soft, smooth texture and a pH of 5.34 ± 0.05. The in vitro release of CUR from the NE-based gel was higher than that from a commercial gel with nanosized CUR (21.68 ± 1.25 µg/cm^2^, 13.62 ± 1.63 µg/cm^2^ after 10 h, respectively). The CUR-NE gel accelerated in vitro antibacterial and in vivo wound healing activities as compared to other CUR-loaded gels. The CUR-NE gel has potential for transdermal applications.

## 1. Introduction

Traditional medicine has historically utilized turmeric powder to treat a number of conditions, including rheumatic diseases, diabetes, cancer, liver diseases, infectious diseases, and digestive problems such as flatulence, dyspepsia, and gastric/duodenal ulcers. Curcumin (CUR) research has revealed a wide range of intriguing biological and therapeutic functions, including anti-microbial, anti-cancer, anti-inflammatory, and anti-diabetic qualities [1]. On the other hand, CUR has low solubility, permeability, and stability, making it difficult to administer the chemical efficiently via the skin. To overcome this issue, new research has looked at the use of nanosystems, such as polymeric nanosystems and liposomes, to increase CUR’s distribution and effectiveness [2].

Nanoparticle-based drug delivery approaches offer promising ways to increase the efficacy of CUR in treating a range of diseases, especially those that are infectious. In multiple in vitro and in vivo studies, CUR nanoparticles have demonstrated greater therapeutic outcomes compared to free CUR. Furthermore, the nano-sized formulation of CUR enhances its solubility in water and boosts its antimicrobial activity [1]. Nanocurcumin, produced from CUR extracted from turmeric rhizome, exhibited greater antibacterial action against *Staphylococcus aureus* (*S. aureus*) and *Escherichia coli* (*E. coli*) bacteria compared to bulk curcumin. The nanocurcumin-loaded cream was more antimicrobial than bulk curcumin cream after a month of storage [3]. Wet-milled curcumin nanoparticles were tested for antibacterial activity against four bacteria by Adahoun M. A. et al. in 2017. Nanocurcumin inhibited all tested bacteria better than bulk curcumin [4]. Because of the potency of CUR’s antibacterial effect, recent research has focused on developing nanocarriers as CUR drug delivery vehicles [1,5]. Numerous studies have incorporated CUR into various nanoformulations, including polymeric nanoparticles [6,7,8], liposomes [9], solid lipid nanoparticles [10], and micelles [11], with the aim of enhancing CUR’s antibacterial efficacy.

Nanoemulsion (NE)-based technologies have received a lot of interest in recent decades for overcoming the stratum corneum barrier for effective transdermal drug delivery. A colloidal system composed of immiscible liquids, that is, oil and water stabilized by emulsifiers, is known as an NE [12]. NEs offer a wide range of possible uses due to their tiny size, including drug delivery, food manufacturing, and cosmetics [13].

NEs have been widely employed for the transdermal administration of hydrophobic and hydrophilic molecules that have difficulties with solubility, lipophilicity, and bioavailability. Nowadays, therapeutic plant formulations might be advantageous because they have biocompatible, wound-healing, and antibacterial qualities. Their use can help to reduce cytotoxicity, improve wound healing, and minimize antibiotic resistance.

Gel networks allow nanovesicles to migrate from the matrix to the skin surface easily and in a controlled manner, and hydrated skin allows for higher payloads. Biocompatible polymers such as Carbomers, Pluronics, xanthan gum, and carrageenan were also employed as gel systems to deliver emulsified drugs transdermally [12].

Piroxicam oil/water (O/W) NE gel was made by Dhawan et al. (2014) from oleic acid, Tween 80, ethanol (Smix: a surfactant and co-surfactant mixture), and water. The marketed product had lower skin retention, greater skin permeation flux, and longer lag time [14]. In 2017, a nanoemulgel outperformed its NE counterpart (18% Capryol 90, 30% OP-10, 15% 1,2-propylene glycol) in terms of triptolide release and pharmacokinetic profile. The gel maintained the drug release due to the presence of 1.5% *w*/*v* Carbomer 940, which provided a 2.93-fold greater systemic circulation AUC than a drug solution [15].

CUR encapsulation in Nes has been studied to improve its pharmacological activities. Ahamad N. et al. selected clove oil, Tween 80, and PEG 400 as excipients because they could dissolve the drug ingredient and create the biggest emulsion area. The NE healed wounds quicker than the control samples [16]. In 2019, Kole et al. prepared o/w NE containing tetrahydroxy CUR by the high-pressure homogenization technique. The results of the antimicrobial activity essay showed good effects against *E. coli* and *Bacillus subtilis* [17]. A novel NE technology was developed in order to evaluate its effectiveness in diabetic rats with open incision wounds. The NE system significantly decreased oxidative stress, expedited collagen deposition, and avoided the bacterial infection of the wound, all of which sped up the regeneration of skin tissue [18]. Low-energy emulsification enhanced the CUR NE, which was gelled with crosslinked polyacrylic acid (Carbopol 934) to form a nanoemulgel. In psoriatic mice, those treated with a nanoemulgel healed faster than those treated with a CUR and betamethasone-17-valerate gel [19]. High-energy ultrasonic emulsification was used to encapsulate CUR in an NE system. A 0.5% Carbopol^®^ 940 hydrogel was used to topically apply the optimized CUR-loaded NE. The CUR nanoemulgel had a thixotropic rheology, increased skin penetrability, and improved the effectiveness of in vivo wound healing [20].

For ease of scaling-up, the goal of this work was to optimize and analyze the physicochemical characteristics of CUR NE formulations fabricated by the phase inversion method. The optimized CUR NE was then introduced into the gel to evaluate its in vitro antibacterial activity and in vivo wound healing properties, as compared to a pure material-loaded gel and a commercial gel containing nanosized CUR.

## 2. Results

### 2.1. The Saturation Solubility of CUR in Different Excipients

Table 1 shows the findings of the assessment of CUR’s solubility in different excipients.

Because of its high solubilizing capacity, Capryol 90 was chosen as the oil phase among the oil excipients. Among the surfactants, Labrasol exhibited the greatest saturation solubility of CUR, followed by the LS:CRH40 mixture. Cremophor RH40 was not chosen since its CUR became dark red during testing. CUR was shown to dissolve better in Lauroglycol as a co-surfactant than in propylene glycol. The findings of the solubility studies would be used in combination with the emulsification study to identify suitable Smix components [21].

### 2.2. Emulsification Efficiency

Table 2 shows the findings of an investigation into the emulsification potential of surfactants and co-surfactants.

Because of their capacity to emulsify oil to generate a clear emulsion, the LS:CRH40 mixture and propylene glycol were selected as the surfactant and co-surfactant, respectively [21].

### 2.3. Construction of Ternary Phase Diagrams

**Figure 1:**
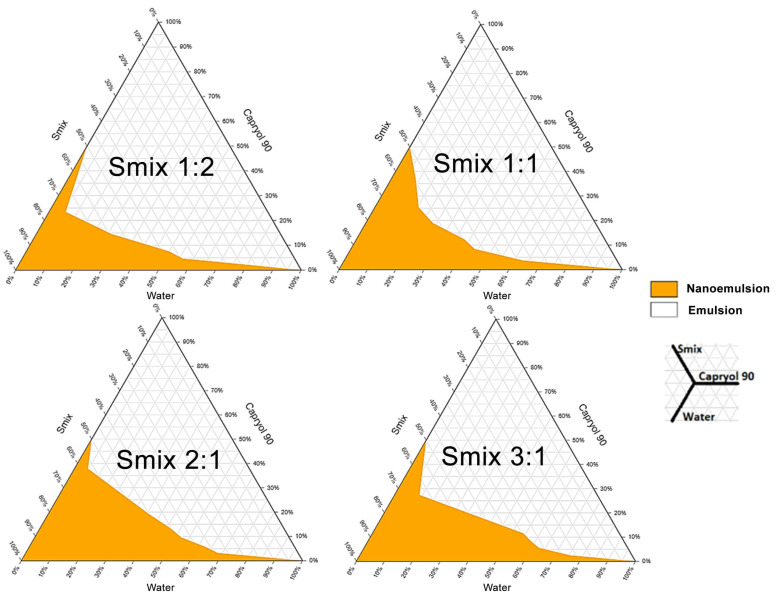
A phase diagram constructed for various Smix (a surfactant and co-surfactant mixture) ratios.

The phase diagrams for various Smix ratios are illustrated in Figure 1 below.

For future investigations, the phase diagram corresponding to Smix 2:1 with the greatest clear emulsion zone was chosen [21].

### 2.4. Optimization of CUR-Loaded NE

Based on the phase diagram of Smix 2:1, the range of variation for the input variables were selected, as shown in Table 3 [21].

The experimental design and the results obtained from the experiments during the optimization process are presented in Table 4 [21].

The translucent emulsion droplets varied in size from 17.73 to 69.12 nm, which was within the acceptable size range of 10–100 nm. The polydispersity index (PDI) values of 0.318–0.658 implied a wide droplet size dispersion [21].

### 2.5. The Effects of Factors on Droplet Size and Droplet Size Distribution

**Figure 2:**
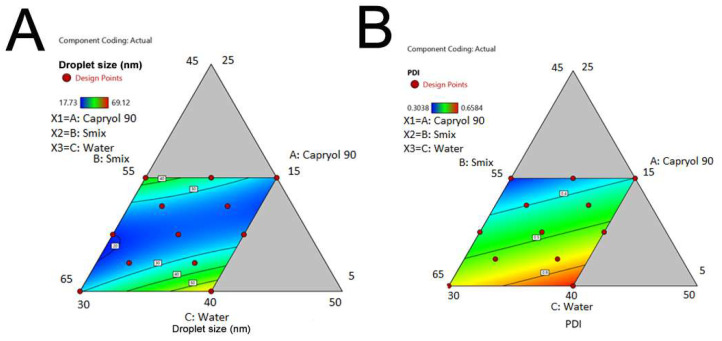
The influence of factors (% Capryol 90, % Smix, % water) on droplet size (**A**) and PDI (**B**) of CUR NE.

Figure 2 shows the effect of input parameters on droplet size and PDI of CUR-NE [21].

In general, droplet size was larger in the region with high-water content and low-oil content, as well as in the region with high-oil content and low-water content. When the oil ratio was 5%, increasing the amount of Smix led to a smaller droplet size, while at a 15% oil ratio, increasing the amount of Smix resulted in a larger droplet size.

Overall, increasing the water ratio resulted in an increase in PDI. With a fixed water ratio, PDI increased as the Smix ratio increased, and the oil ratio decreased. Similarly, when the oil ratio was fixed, an increase in the water ratio led to an increase in PDI.

The equations used to predict the relationship between droplet size, PDI, and the input variables, along with the statistical parameters of the prediction models, are shown in Table 5 [21]:

Y1 = 199.06X1 + 29.29X2 + 89.76X3 − 276.14X1X2 − 473.96X1X3(1)

Y2 = 0.0685X1 + 0.5631X2 + 0.7334X3(2)

Based on the results, it can be observed that the F-value of Equation (1) is 4.35, and the *p*-value is 0.0443, indicating that the prediction model was significant. The negative value of R^2^_pre_ suggests that the overall mean value could be better predicted for the response to droplet size than the current model. Moreover, Equation (2) has an F-value of 10.55 and a *p*-value of 0.0044, demonstrating that the prediction model was significant. The difference between R^2^_adj_ and R^2^_pre_ was less than 0.2, indicating the validity of the model.

The optimized formula had a desired coefficient of 0.798 with X1 (% Capryol 90) at 11.83%, X2 (% Smix) at 58.17%, and X3 (% water) at 30%. For the output variables (Y1, Y2), the expected values were 25.17 nm and 0.384, respectively. The actual examination findings were 22.87 nm and 0.348, respectively. The droplet size and PDI were both within the predicted ranges (droplet size, 10–100 nm, and PDI, <0.5).

### 2.6. Characterization of CUR-Loaded NE and CUR-NE Gel

**Figure 3:**
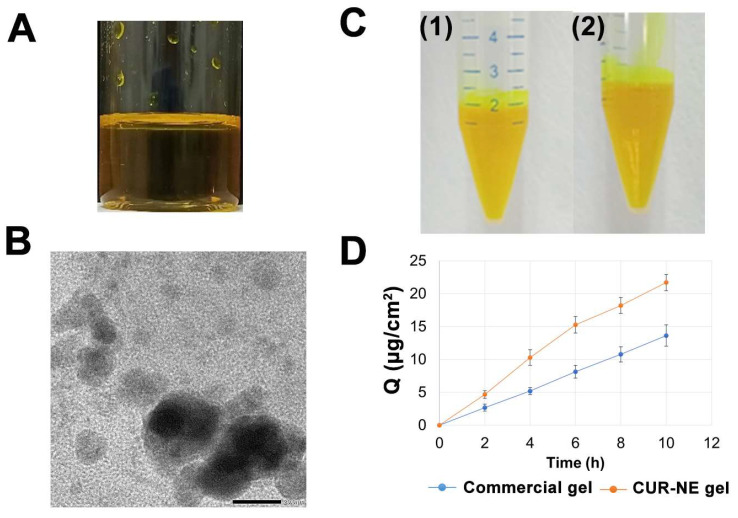
Visual appearance (**A**), TEM image ((**B**), scale bar of 20.0 nm), stability of CUR-NE before and after centrifugation ((**C1**) and (**C2**), respectively), and cumulative drug release of CUR-NE gel (**D**) (CUR-NE: Curcumin nanoemulsion).

The physicochemical properties of the optimized NE were evaluated, and the results are displayed in Figure 3 [21].

The obtained CUR-NE was yellow-orange in color, transparent, and homogeneous. The TEM images indicated spherical particles with sizes ranging from 15 to 25 nm, with some particles clumping together. The emulsion was stable with no phase separation and no drug precipitates after centrifugation, satisfying the kinetic stability criteria.

Conforming to the proposed standard, the emulsion contained 0.518 ± 0.016% CUR, corresponding to 103.52 ± 3.17% of the labeled content (0.5%).

When compared to the commercial gel, the CUR-NE gel released more drug amounts at each sample time point. After 10 h, the cumulative amount of CUR in CUR-NE gel was 21.68 ± 1.25 g/cm^2^, which was 1.59 times greater than that of the commercial gel at 13.62 ± 1.63 g/cm^2^. This difference was statistically significant (*p* < 0.05, n = 3).

Additionally, the CUR-NE gel was soft, smooth with a yellow color and had a pH of 5.34 ± 0.05, which is in line with the physiological pH of the skin (4.2–5.6), thus minimizing the risk of irritation [21].

### 2.7. In Vitro Antimicrobial Activity

**Figure 4:**
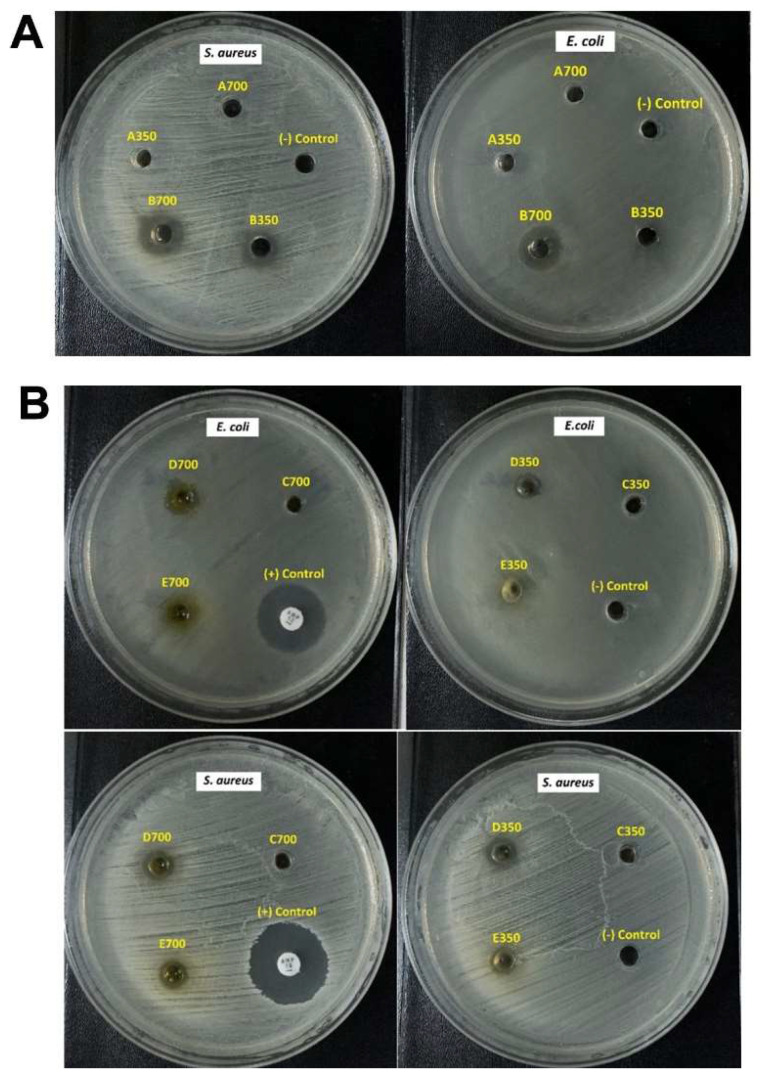
(**A**) Antibacterial activity of CUR aqueous suspension (A700, A350), CUR NE (B700, B350) and placebo (as (−) Control); (**B**) Antibacterial activity of CUR gel (C700, C350), CUR-NE-based gel (D700, D350), Commercial gel (E700, E350), Ampicillin (as (+) Control) and placebo (as (−) Control).

The antibacterial efficacies of the various samples were displayed in Table 6 and Figure 4. Ampicillin (10 µg), used as positive control, demonstrated the maximum zones of inhibition against *S. aureus* and *E. coli* (21.0 mm and 18.4 mm, respectively). The aqueous suspension of CUR and the CUR gel did not exhibit any efficacy against the tested bacteria at the tested concentrations. The commercial gel displayed antibacterial activity against *S. aureus*, whereas its efficacy against *E. coli* was only observed at a high concentration (700 µg/mL). In contrast, the CUR NE showed potential antibacterial efficacy against *S. aureus* and *E. coli* at both concentrations. Furthermore, the inhibition zone results also showed the antibacterial efficacy of the CUR-NE-based gel against both tested bacteria.

### 2.8. In Vivo Wound Healing Study

**Figure 5:**
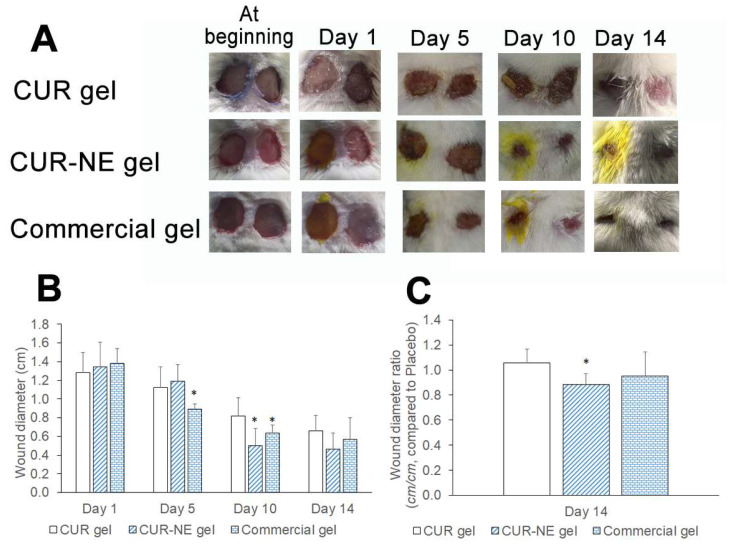
(**A**) Imaging of circular wound areas at days of treatment of CUR gel, CUR-NE gel, and a commercial gel with nanosized CUR (Left wounded area: treatment with different CUR-loaded gels and right wounded area: Placebo gel treatment); (**B**) The change in wound diameter after treatment with different CUR-loaded gels and (**C**) The wound diameter ratio (cm/cm) of groups treated with different CUR-loaded gels and Placebo gel at day 14 (*: *p* < 0.05 vs. mice treated with CUR gel, n = 6).

Figure 5 displays the variations in the size of the wounds. In comparison to the control groups, mice treated with the CUR-loaded NE-based gel showed increased wound healing. Based on the obtained macroscopic findings, it was found that the CUR-loaded NE-based gel was almost more potent than the commercial formulation and the formulation containing pure CUR.

### 2.9. Histopathological Study

**Figure 6:**
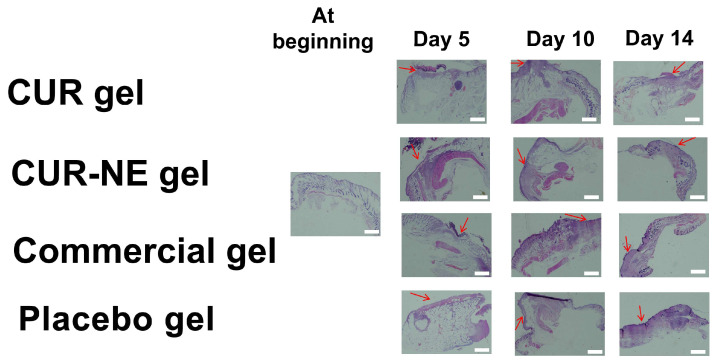
H&E staining wounded tissues of CUR-treated and Placebo gel groups after 14-day treatment: CUR gel; CUR-NE gel; Commercial gel (with nanosized CUR); Placebo gel (Red arrows: wounded regions, scale bar of 1 mm).

The CUR formulation improved epidermal and dermal regeneration, according to histological studies (Figure 6). Re-epithelization was found in mice in the formulation-treated group after 5 days of therapy. When re-epithelization and wound healing were examined in the 14-day groups, the CUR-NE gel and commercial gel groups performed significantly better than CUR gel group and Placebo gel group. Compared to the commercial group, the CUR-NE gel group produced much superior outcomes. In all groups, wound healing was observed. The CUR-NE gel formulation was found to enable faster re-epithelization and wound healing.

## 3. Discussion

The investigation of a drug’s solubility in excipients is necessary to select the components for the NE formula, especially for the oil phase. For poorly water-soluble drugs, such as CUR, the drug will mostly distribute in the oil phase. Therefore, an oil phase that can dissolve CUR well will help increase the drug-loading capacity of the NE [22]. Additionally, evaluating the emulsification ability is an important basis for selecting suitable surfactants and co-surfactants to be included in the formula. This is a key test to ensure that NE meets the prerequisite requirements of uniformity and clarity, especially for NEs prepared using low-energy methods. Meanwhile, constructing a phase diagram is a method used to roughly determine the relationship between the ratios of the three basic components in the formula, including oil, Smix, and water, to form the emulsion [23].

Initial investigations help determine the appropriate range of variation of the three components, which is the region that forms the emulsion on the phase diagram, as well as to determine the water content needed to direct it into a hydrogel form. Droplet size and PDI are two basic parameters for evaluating nanoscale particles and their distribution, respectively, with droplet size playing an important role in the stability and skin penetration ability of the NE [12]. Therefore, they were selected as output variables to optimize the NE’s preparation formula. The droplet size obtained was uniformly within the size range of the NE (10–100 nm), while PDI < 0.5 indicated that the size distribution was not substantial. These results were supported by the study of Ahmad et al. (2019) on CUR-NE prepared using ultrasonication with droplet size range of 50.85 to 188.60 nm and PDI of 0.256 to 0.559 [16].

Regarding the effect of input variables on droplet size, when the water ratio was high and the amount of oil was low, as well as for the lowest water content region and the highest oil content region, the droplet size was significantly larger, similar to the study of Kumar N. et al. (2015) [24]. In addition, the prediction equations of Kumar N. et al. (2015) and Fouad S. A. et al. (2013) showed that the independent variables of oil, water, and Smix ratios increased droplet size, while the interaction between these variables decreased droplet size. Equation (1) in this study also yielded similar results [24,25].

When the oil ratio was fixed at its lowest level (5%), more Smix amount led to smaller droplet sizes. When the oil level was at 15%, more Smix amount led to larger droplet sizes. This could be explained by the fact that a greater amount of Smix could help to emulsify the oil well, reducing the size of the oil droplets, but when the oil ratio was high, the Smix no longer effectively emulsified [24].

Regarding the influence of input variables on PDI, Equation (2) showed that all independent factors increased the distribution of droplet size. As the water ratio increased, the interfacial surface area between the oil and water phases increased. This could reduce the emulsifying ability of Smix and result in the production of O/W emulsion droplets with less uniform sizes. Meanwhile, when the Smix ratio was too high, it could create micelles or aggregates due to excess surfactant molecules. O/W emulsion droplets with different surfactant concentrations at the interfacial layer could also be formed, leading to non-uniform sizes [26]. With a relatively high desirability coefficient of 0.798, the optimized formula included 11.13% oil, 58.87% Smix, and 30% water. This composition could ensure the formation of an O/W emulsion that was suitable for application in hydrogel form for external use, which was superior to the formulation reported by Fouad et al. (2013) [25].

The O/W emulsion met appearance requirements, did not undergo phase separation after centrifugation, and had a spherical shape as observed by TEM. Incorporating the O/W emulsion into a gel form with Carbopol showed better in vitro drug release performance compared to a commercial gel product containing nanosized CUR (with an equivalent amount). This suggests that the O/W emulsion contributed to enhancing the solubility and dissolution rate of the drug, as well as improving its diffusion and permeability properties. Therefore, this drug delivery system has potential applications in gel forms for increasing the bioavailability of CUR for topical use.

The present study assessed the antibacterial efficacy against *S. aureus* and *E. coli* of CUR NE, CUR-NE-based gel through the agar well diffusion method. In most of the tested concentrations of samples (CUR NE, CUR-NE-based gel, commercial gel), the antimicrobial effects against Gram-positive bacteria (*S. aureus*) were greater than those against Gram-negative bacteria (*E. coli*), which may be due to the different compositions and structures of bacterial cells. This outcome was similar to the reported study by Asabuwa Ngwabebhoh F. et al. (2018) [27]. Comparing CUR NE and CUR aqueous suspension, CUR NE showed potential antibacterial efficacy against Gram-positive bacteria (*S. aureus*) and Gram-negative bacteria (*E. coli*) at both concentrations, whereas CUR aqueous suspension had no antibacterial efficacy to any of tested bacteria species. This could be attributed to the increased diffusion capacity of CUR in NE droplets into the agar medium and microbial membrane as compared to that in the aqueous suspension, due to their smaller size. Because of its ability to successfully penetrate into the bacterial cell wall, CUR being encapsulated in an oil phase droplet was able to perform antibacterial activity. This led to the lysis of the peptidoglycan layer, which ultimately resulted in a deformation and lysis of the bacterial cell [18]. Antibacterial activities of CUR could be due to its interaction with the cell division-initiating protein FtsZ, which was related to its methoxy and hydroxyl groups [28]. The result confirmed that oil-in-water NE is a potent delivery system to improve the antibacterial activity of CUR.

In the case of the CUR-NE-based gel, the diameters of its inhibition zones were smaller than the CUR NE’s. This could be explained by the elevated viscosity of the gel compared to the NE. However, the observations implied that antibacterial efficacy was maintained when the NE was transformed into gel form. On the other hand, the CUR-NE-based gel showed somehow efficacy against tested bacteria than commercial gel while the CUR gel showed no effect at the tested CUR concentrations of 350 or 700 µg/mL. The results indicated that the CUR-NE-based gel had more antibacterial activity than the commercial gel with nanosized CUR and enhanced the antibacterial property compared to the conventional gel. Additionally, the antibacterial effectiveness of CUR encapsulation in NE assisted in lowering the bacterial load and improving wound healing [29].

Wound healing is known to be a complicated and dynamic process that generally contains discrete phases denoting the healing stages and necessitates the participation of various cell types in a range of cellular activities [30]. Hemostasis is the initial step of wound healing, followed by the inflammation phase, the proliferative phase, and the maturation phase. The inflammation phase is a critical and required step for wound healing. During this phase, cells associated with inflammation (neutrophiles, macrophages, and so on) move to the wound site and ensure the removal of germs and tissue remnants from the environment. Fibroblasts and epithelial cells are also stimulated by inflammatory cells [31]. Both acute and chronic forms of inflammation may exist. Acute inflammation helps the wound heal; however, chronic inflammation slows down the healing process and makes it take longer [32]. CUR was demonstrated in previous studies to have wound healing, antibacterial, and anti-inflammatory effects [3,33]. The wound healing of CUR is also attributed to its antioxidant activity [34,35]. In the study of Ouyang et al., the results indicated that the free radical scavenging ability of the novel multifunctional hydrogel promoted hemostatic function in wound management [36]. The antioxidant activities of CUR were protected in CUR-loaded NE and CUR-NE gels [35,37]. When the findings of the wound healing trial were analyzed, it revealed that the CUR-NE-based gel formulation had greater effectiveness than the CUR gel and commercial gel groups. The tissue regeneration in the epithelial and dermal tissues was more considerable than in the CUR gel and commercial gel groups, particularly in histopathological alterations. Histological tests revealed that the CUR-NE-based gel formulation enhanced re-epithelization and vascularization in injured tissue, hence expediting wound healing. Recovery was also quicker when the shrinking of the wound region’s diameter was assessed [38]. For further usage in clinical trials, the biocompatibility of CUR NE and CUR-NE gel should be evaluated [39,40]. However, in this study, excipients (Capryol 90, Labrasol, Cremophor RH40, propylene glycol, Carbopol 940, etc.) used in the investigated formulations for preparing topical CUR-NE gels were used in approved pharmaceutical products and/or are generally recognized as safe (GRAS) by the Food and Drug Administration [41,42].

## 4. Materials and Methods

### 4.1. Materials

CUR was purchased from India (purity of 95%). Standard CUR was obtained from the Vietnam Institute of Quality control (99.56%). Capryol 90, Labrafil, Labrasol, and Lauroglycol were obtained from Gattefossé (Paris, France). All other chemicals were of analytical grade. A commercial gel (containing nanosized CUR) is a marketed product, which was purchased at a pharmacy in Vietnam.

### 4.2. Methods

#### 4.2.1. Assessment of the Saturation Solubility of CUR in Different Excipients

The saturation solubility of CUR was investigated in several excipients, including oils (Capryol 90 and oleic acid), surfactants (Labrafil, Labrasol, and Cremophor RH40), and co-surfactants (propylene glycol, Lauroglycol). A centrifuge tube with 1 mL of excipient was agitated to disperse excess CUR. To reach equilibrium, the tube was placed in a thermostatically controlled bath shaker at 37 °C and 100 rpm for 72 h. The tube was centrifuged at 10,000 rpm (Hermle Z32 HK, Wehingen, Germany) for 10 min. Supernatant was collected and filtered using a 0.45 µm membrane filter. To estimate CUR saturation solubility in excipients, the sample was diluted in methanol and analyzed using a UV-Vis spectrophotometer at 421 nm (Jasco V-630, Tokyo, Japan).

#### 4.2.2. Evaluation of Emulsification Efficiency

The water emulsification ability of 0.1 g of each excipient (Labrafil, Labrasol, LS:CRH40 mixture, propylene glycol, and Lauroglycol) was tested in 2 mL of distilled water. Then, 10 µL of oil was progressively added to the solution while stirring and recorded. The final point occurred when a turbid emulsion developed [19].

#### 4.2.3. Construction of Ternary Phase Diagrams

Water titration was employed on selected excipients to construct phase diagrams. The procedure required making Smix blends (surfactant/co-surfactant) with ratios of 3:1, 2:1, 1:1, and 1:2 (*w*/*w*). Each Smix combination had oil/Smix ratios of 0.5:9.5, 1:9, 1.5:8.5, 2:8, 2.5:7.5, 3:7, 4:6, and 5:5 (*w*/*w*). While stirring, water was progressively added to each oil/Smix mixture until the clear emulsion became cloudy or turbid. Using ternaryplot.com’s free tool, phase diagrams were created using each emulsion’s oil, Smix, and water percentages.

#### 4.2.4. Optimization of CUR-Loaded NE

The Smix mixture was created by mixing the surfactant and co-surfactant in proper proportions. The oil was added and agitated until it produced a uniform oil phase. CUR was added to the oil phase (at a concentration of 0.5% in NE formulations) and stirred until fully dissolved. Distilled water was gently added to the mixture and magnetically agitated to create a uniform emulsion [19].

The D-optimal model with 12 tests was designed using Design Expert 12.0 software. The three primary excipient system components—oil, Smix, and water—were the input variables (X1, X2, X3, respectively). Based on the phase diagram analysis, the oil and Smix components and ranges were identified. Y1, the droplet size (Size), and Y2, the PDI, were the output variables. The optimization was run using Design Expert 12.0 software with the following settings: 3 input variables with “in range” variation, and Y1 and Y2 of minimum values with equal weight.

#### 4.2.5. Formulation of CUR-NE-Based Gel

Carbopol 940 was dispersed in water to form a gel by swelling overnight and neutralized with triethanolamine. The gel was homogeneously mixed with the NE using a magnetic stirrer [19]. The components were mixed in appropriate proportions to achieve the final gel formula containing 0.035% (*w*/*w*) of CUR in 1% (*w*/*v*) of Carbopol 940.

### 4.3. Characterization of CUR-Containing NE

#### 4.3.1. Appearance

The NE was yellow to orange, clear, and homogenous. Any cloudiness, phase separation, active component crystallization, or discoloration was noted.

#### 4.3.2. Droplet Size and Size Distribution

A diluted NE sample was prepared at a suitable ratio to achieve a counting rate of 200–400 kcps. A plastic cuvette was used to measure the sample using a Zetasizer Lab device (Malvern, UK).

#### 4.3.3. Particle Morphology

A high-resolution transmission electron microscope (HR-TEM, JEM 2100, Jeol, Tokyo, Japan) was used to examine the morphology of the CUR NE.

#### 4.3.4. Dynamic Stability

The centrifugation technique was used to assess dynamic stability. Two milliliters of the emulsion was placed in a Falcon tube and centrifuged for 30 min at 5000 rpm (Hermle Z32 HK, Wehingen, Germany) [43].

#### 4.3.5. Assay for Drug Content

The concentration of CUR was measured using UV-visible spectrophotometric analysis (Jasco V-630, Tokyo, Japan) with a wavelength of maximum absorbance at 421 nm after being diluted with methanol (1:1000) (Figures S1–S3) [21].

### 4.4. Characterization of CUR-NE Gel

#### 4.4.1. Visual Appearance and pH

The appearance, the presence of particle matter, and the homogeneity of gel formulations containing CUR-NE were visually assessed. The pH of each hydrogel formulation (1 g) was determined using a pH meter (pH Sension PH3, HACH, Loveland, CO, USA) [44].

#### 4.4.2. In Vitro Drug Release

Hanson Research’s release device with a receptor volume of 7.0 mL and a working surface of 1.767 cm^2^ was used for in vitro drug release. A dialysis cellulose membrane (12–14 kDa, Visking tube, Medicell, London, UK) was used as the releasing membrane. After equilibration with the receptor medium (ethanol and distilled water in a 1:1 (*v*/*v*) ratio) for 1 h, 0.3 g of CUR-loaded NE gel was gently applied to the membrane. The control gel used was a commercial gel. The release medium was maintained at 37 ± 0.5 °C and 350 rpm. At set times, 2 mL aliquots were removed and replaced with the same amount of fresh medium [40]. The CUR concentration in each sample was measured using UV-visible spectrophotometry (Jasco V-630, Tokyo, Japan) at a wavelength of maximum absorbance of 431 nm (Figure S4) [21].

#### 4.4.3. In Vitro Antimicrobial Activity

The antibacterial efficacies of CUR NE and CUR-NE-based gel were tested against *S. aureus* ATCC 25923 (Gram-positive bacteria) and *E. coli* ATCC 25922 (Gram-negative bacteria) (MicroBiologics, St Cloud, MN, USA). The agar well diffusion method was processed based on the study of Hettiarachchi with some modifications [3]. Before the experiments, Petri dishes were prepared by pouring Mueller–Hinton (MH) agar (Merck, Darmstadt, Germany) and allowing it to solidify. The bacterial cultures were adjusted to 0.5 McFarland standard by diluting with 0.9% sterile sodium chloride solution [45]. The suspensions of tested bacteria were swabbed on the surface of MH agar plates using a sterilized cotton swab to maintain uniform distribution of the bacteria across the plate surface. Then, wells were created by using the sterile back of a Pasteur pipette. The samples (CUR NE, CUR aqueous suspension (CUR), CUR-NE-based gel, CUR gel, Commercial gel) were dispersed in distilled water at a CUR concentration of 700 µg/mL or 350 µg/mL. After that, about 50 µL of prepared suspensions were added into wells. Placebo (A vehicle without CUR) was used as a negative control. The standard antibiotic disc containing Ampicillin (10 µg, Liofilchem srl, Roseto degli Abruzzi (TE), Italy) served as a positive control for both *S. aureus* and *E. coli*. The plates were then incubated at 37 °C for 24 h. After the incubation, inhibition zones were determined by measuring their diameters using a caliper (RS PRO, Shanghai, China).

#### 4.4.4. In Vivo Wound Healing Study

The Swiss albino mice (weight; 25–30 g) were housed in cages of six, given unrestricted access to food and water, and kept at a room temperature of 25.0 ± 2.0 °C with natural light/dark cycle. The mice were anesthetized with xylazine (8 mg/kg, Xyla, Interchemie, Venray, The Netherlands) and Zoletil (64 mg/kg, Zoletil 100, Valdepharm, Val-de-Reuil, France) by intraperitoneal injection. The mice’s dorsal hairs were shaved. Two circular incisions (10 mm each) were made on the dorsal interscapular area of each animal by excising the skin using surgical scissors. The following categories make up the animal treatment groups (treated with CUR every 2 days, six mice in each group) for the left wound on each mouse: group CUR-NE gel (CUR-loaded NE-based gel formulation treatment), group CUR gel (CUR-loaded gel treatment), and group Commercial gel (commercial gel treatment). For the right wound on each animal, all groups were treated with a vehicle (placebo gel treatment) every 2 days. The camera was used to take pictures documenting the wound’s progression at the beginning, middle, and end of therapy for 14 days. ImageJ software (version 1.54d, NIH, Bethesda, MD, USA) was used to determine the level of wound closure by measuring the wound diameter and the wound diameter ratio (cm/cm) of groups treated with different CUR-loaded formulations, and Placebo gel at day 14 was calculated [38]. The in vivo experiments were approved by the Institutional Ethics Committee of University of Medicine and Pharmacy, Hue University (Approval number: H2022/034 date on 20 May 2022).

#### 4.4.5. Histopathological Study

On the 5th, 10th and 14th days of the research, the mice were put under anesthesia, and the wound area was removed for histological investigation. Hematoxylin and eosin staining (H&E) was used to stain the extracted tissues. A microscope (Nikon SMZ745T, Tokyo, Japan) was used to identify histopathologic alterations in the dermis and epidermis [38].

#### 4.4.6. Statistical Analysis

To undertake the statistical analysis, the Origin 9.0.0 (MA, USA) software was utilized. The data were displayed as mean ± standard deviation (SD) and analyzed using the Student’s *t*-test or one-way ANOVA followed by Tukey’s multiple comparisons test. Statistical significance was defined as *p* < 0.05.

## 5. Conclusions

This study reported the successful preparation of an NE containing CUR using the emulsion inversion method with the following optimal formula: 0.5% CUR, 11.77% Capryol 90, 19.29% Labrasol, 19.29% Cremophor RH40, 19.29% propylene glycol, and water by using the Design Expert 12.0 software. The resulting NE had a yellow-orange color and was transparent, uniform, and stable throughout the centrifugation with a droplet size of 22.87 nm, and a PDI of 0.348. The TEM analysis showed that the particle shape was spherical. Furthermore, when incorporated into a gel, the CUR-NE gel showed a smooth texture, a pH of 5.34 ± 0.05, and better in vitro drug release capability as compared to a commercial gel with nanosized CUR (21.68 ± 1.25 µg/cm^2^, 13.62 ± 1.63 µg/cm^2^ after 10 h, respectively). The CUR aqueous suspension and CUR gel did not show any antibacterial efficacy against *S. aureus* and *E. coli* at the tested concentrations of 350 and 700 µg/mL. However, the CUR NE and CUR-NE gel showed better antibacterial activity than the CUR gel and commercial gel. Our CUR-NE gel formulation significantly accelerated wound healing in vivo as compared to the CUR gel and commercial gel with nanosized CUR. Therefore, the CUR-containing NE has great potential for use in the development of topical gels to improve the bioavailability of CUR.

## Figures and Tables

**Table 1:** Saturation solubility of CUR in different excipients.

Excipient Type	Excipient	CUR Solubility (mg/g) (Mean ± SD, n = 3)
Oils	Oleic acid	0.17 ± 0.01
	Capryol 90	6.24 ± 0.37
Surfactants	Labrafil	2.19 ± 0.42
	Labrasol	52.49 ± 4.07
	Cremophor RH40	42.95 ± 2.54
	LS:CRH40 (1:1)	48.11 ± 3.42
Co-surfactants	Propylene glycol	0.97 ± 0.09
	Lauroglycol	3.38 ± 0.18

Abbreviation: CUR: Curcumin; LS: Labrasol; CRH40: Cremophor RH40; Labrafil: Labrafil M1944CS; Lauroglycol: Lauroglycol 90.

**Table 2:** Emulsification capability of surfactants and co-surfactants.

Excipients		V_Capryol 90_ (µL) (Mean ± SD, n = 3)	Emulsification Capability
Labrafil	Surfactant	10.00 ± 0.00	Partial emulsification
Labrasol	Surfactant	53.33 ± 5.77	Complete emulsification
LS:CRH40	Surfactant	90.00 ± 10.00	Complete emulsification
Propylene glycol	Co-surfactant	26.67 ± 5.77	Partial emulsification
Lauroglycol	Co-surfactant	13.33 ± 5.77	Partial emulsification

**Table 3:** The input and output variables for optimization of CUR-loaded NE.

Input Values	Symbol	Variation Range	
		Low Level (%)	High Level (%)
% Capryol 90	X1	5	15
% Smix	X2	45	65
% water	X3	30	40
Constraint condition		X1 + X2 + X3 = 100%	
**Output values**		**Constraints**	
Size (nm)	Y1	Minimum	
Polydispersity index (PDI)	Y2	Minimum	

**Table 4:** The physicochemical results of the designed experiments.

No.	X1 (%)	X2 (%)	X3 (%)	Size (nm)	PDI
1	15	50	35	32.91 ± 0.48	0.318 ± 0.016
2	7.5	55	37.5	17.73 ± 1.98	0.552 ± 0.149
3	12.5	55	32.5	33.81 ± 2.54	0.425 ± 0.030
4	15	45	40	27.96 ± 0.81	0.384 ± 0.039
5	10	60	30	26.03 ± 1.01	0.482 ± 0.049
6	12.5	50	37.5	26.94 ± 1.43	0.452 ± 0.061
7	5	55	40	69.12 ± 9.92	0.622 ± 0.287
8	7.5	60	32.5	20.65 ± 0.32	0.658 ± 0.115
9	5	65	30	28.62 ± 4.10	0.475 ± 0.018
10	10	50	40	20.77 ± 0.27	0.592 ± 0.067
11	10	55	35	28.04 ± 2.42	0.396 ± 0.018
12	15	55	30	39.02 ± 0.69	0.338 ± 0.014

**Table 5:** The equations and statistical parameters of predicted models.

Output Variables	R^2^	R^2^_adj_	R^2^_pre_	F-Value	*p*-Value
Y1 (Size)	0.7130	0.5490	−0.9963	4.35	0.0443
Y2 (PDI)	0.7009	0.6345	0.4709	10.55	0.0044

**Table 6:** Inhibition zone of different formulations containing CUR.

Tested Bacteria	Inhibition Zone (mm)										
	CUR		CUR-NE		CUR Gel		CUR-NE Gel		Commercial Gel		Ampicillin
	350 µg/mL	700 µg/mL	350 µg/mL	700 µg/mL	350 µg/mL	700 µg/mL	350 µg/mL	700 µg/mL	350 µg/mL	700 µg/mL	10 µg
*S. aureus*	N	N	7.5 ± 0.4	9.5 ± 0.5	N	N	6.6 ± 0.1	7.8 ± 0.5	6.3 ± 0.1	6.5 ± 0.1	21.0 ± 0.1
*E. coli*	N	N	6.5 ± 0.1	10.8 ± 0.4	N	N	6.2 ± 0.3	8.6 ± 0.4	N	6.2 ± 0.1	18.4 ± 0.1

N: no inhibition zone was found.

## Data Availability

The data presented in this study are available in this article or Supplementary material.

## Supplementary Materials

The following supporting information can be downloaded at: https://www.mdpi.com/article/10.3390/molecules28176433/s1, Figure S1: UV-Vis spectrum of standard curcumin in methanol; Figure S2: UV-Vis spectrum of pure curcumin in methanol; Figure S3: UV-Vis spectrum of CUR gel in methanol; Figure S4: UV-Vis spectrum of curcumin in in vitro release media.
