# Supplementary material for: Development and Characterization of a Hydrogel Containing Curcumin-Loaded Nanoemulsion for Enhanced In Vitro Antibacteria and In Vivo Wound Healing
Source: Molecules. 2023 Sep 4;28(17):6433. doi: 10.3390/molecules28176433 (PMC10490385; doi:10.3390/molecules28176433)
Supplement: Supplementary file 1 [file molecules-28-06433-s001.zip › molecules-2576097-supplementary.pdf]

Supplementary Materials

Development and Characterization of a Hydrogel Containing Curcumin-Loaded Nanoemulsion for Enhanced In Vitro Antibacteria and In Vivo Wound Healing

Thi Thanh Ngoc Le [1], Thi Kieu Nhi Nguyen [1], Van Minh Nguyen [2], Thi Cam Minh Dao [1], Hoai Bao Chau Nguyen [1], Cong Thuan Dang [3], Thi Bao Chi Le [4], Thi Khanh Linh Nguyen [4], Phuong Thao Tien Nguyen [3], Le Hoang Nam Dang [5], Van Minh Doan [6] and Hoang Nhan Ho [1,*]

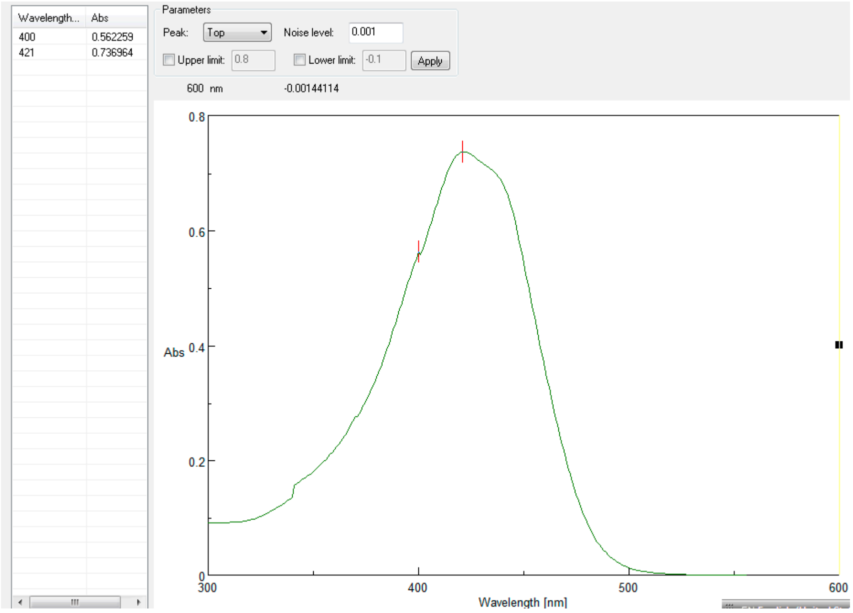

Figure S1. UV-Vis spectrum of standard curcumin in methanol

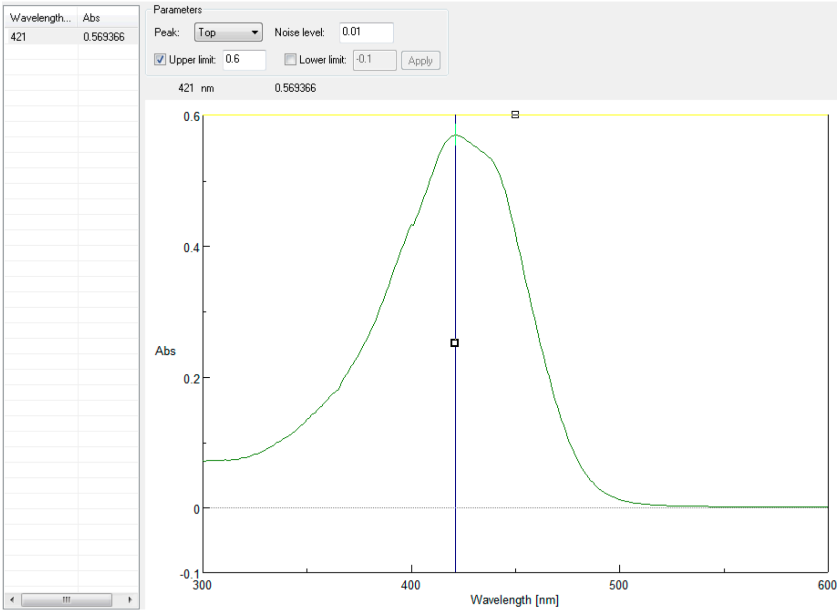

Figure S2. UV-Vis spectrum of curcumin material in methanol

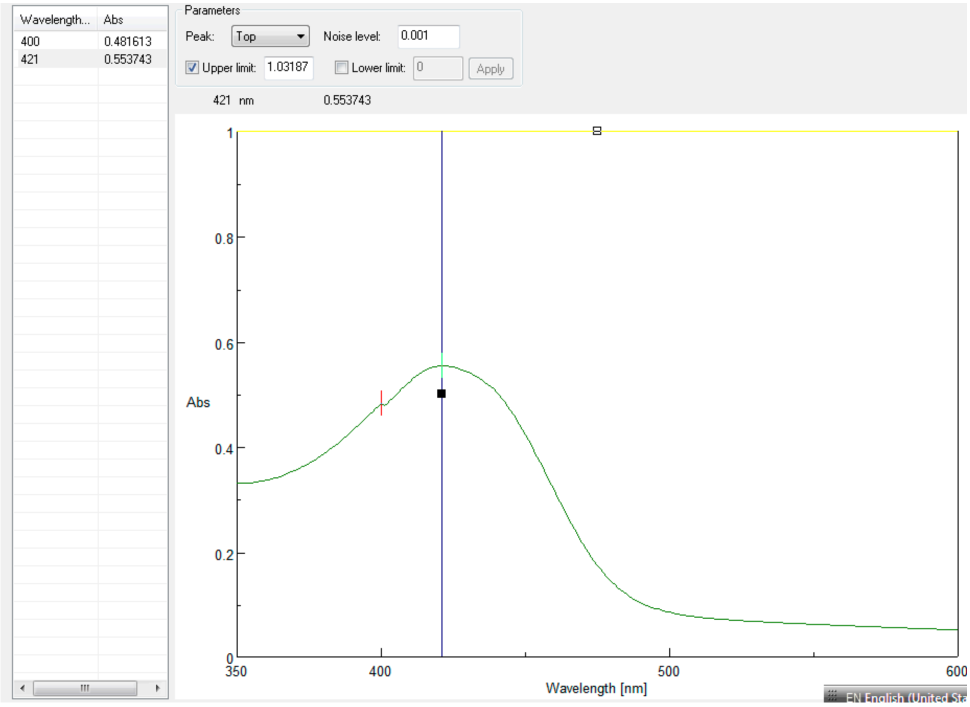

Figure S3. UV-Vis spectrum of a commercial gel containing nanosized curcumin in methanol

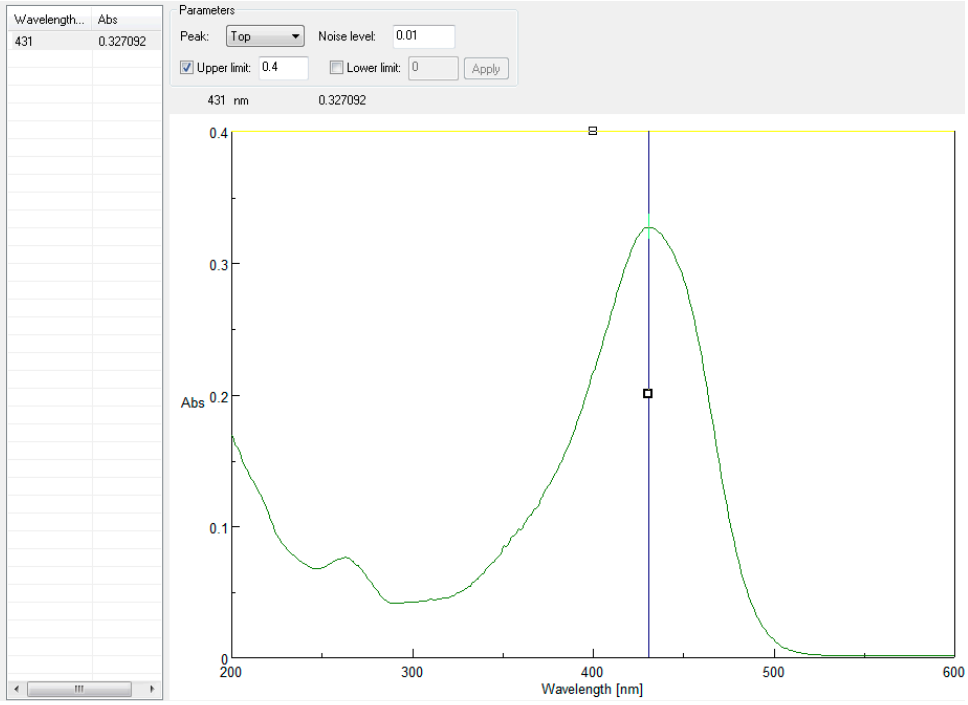

Figure S4. UV-Vis spectrum of curcumin in *in vitro* release media
